# A protocol for feasibility of plasma based GeneXpert platform and Dried Blood Spot (DBS) based Abbott platform for HIV-1 viral load testing among the people living with HIV attending ART centers in India

**DOI:** 10.1371/journal.pone.0285942

**Published:** 2023-05-18

**Authors:** Suchit Kamble, Pallavi Shidhaye, Anupam Mukherjee, Madhuri Tak, Shilpa Bembalkar, Sumit Aggarwal, Arpita Deb, Neha Kapoor, Vinita Verma, Chinmoyee Das, Smita Kulkarni, Manisha Ghate, Sheela Godbole

**Affiliations:** 1 ICMR-National AIDS Research Institute, Pune, India; 2 Indian Council of Medical Research, New Delhi, India; 3 National AIDS Control Organization, New Delhi, India; Stellenbosch University, SOUTH AFRICA

## Abstract

**Background:**

HIV-1 Viral load (VL) measures efficiency of the antiretroviral therapy (ART) after treatment initiation and helps to diagnose virological failures at an early stage. Current VL assays require sophisticated laboratory facilities. As well as there are other challenges pertaining to insufficient laboratory access, cold-chain management and sample transportation. Hence the number of HIV-1 VL testing laboratories is inadequate in the resource limited settings. The revised national tuberculosis elimination programme (NTEP) in India has developed a vast network of point of care (PoC) testing facilities for diagnosis of tuberculosis and several GeneXpert platforms are functional under this programme. Both the GeneXpert HIV-1 assay and HIV-1 Abbott real time assay are comparable and GeneXpert HIV-1 assay can be used as PoC for HIV-1 Viral load testing. Also, the dried blood spot (DBS) as a sample type has been considered as a good option for HIV-1 VL testing in hard to reach areas. This protocol is therefore developed to assess the feasibility of integrating HIV-1 VL testing among people living with HIV (PLHIV) attending ART centres using the two public health models under the current programme: 1. HIV-1 VL testing using GeneXpert platform and plasma as a sample type, and 2. HIV-1 VL testing using Abbott m2000 platform and DBS as a sample type.

**Methods:**

This ethically approved feasibility study will be implemented at two moderate to high burden ART centres where VL testing facility is not available in the town. Under Model-1, arrangements will be made to carry out VL testing on the adjacent GeneXpert facility and under Model-2, DBS will be prepared on site and couriered to identified viral load testing laboratories. In order to assess the feasibility, data will be collected on pretested questionnaire pertaining to number of samples tested for VL testing, number of samples tested for tuberculosis (TB) diagnosis and the turnaround time (TAT). In-depth interviews will be conducted among the service providers at ART centre and different laboratories for addressing any issues regarding the model implementation.

**Results:**

The proportion of PLHIV tested for VL at ART centres, total TAT for both models including TAT for sample transportation, sample testing and receipt of results as well as proportion of sample rejections and reasons for the same, correlation coefficient between DBS based and plasma based VL testing will be estimated using various statistical tools.

**Conclusion:**

If found promising, these public health approaches will be helpful for the policy makers and program implementation in scaling up HIV-1 viral load testing within India.

## Background

There are around 37.7 million people living with HIV (PLHIV) across the globe and 27.5 million are on antiretroviral therapy (ART) (2020) [1]. Improved access for ART has reduced morbidity and mortality and also improved quality of life among PLHIV. With estimated adult HIV prevalence of 0.21% (2021), there are 2.401 million PLHIV in India, out of which 1.38 million PLHIV are on ART. In India, 78% PLHIV were knowing their HIV status, 83% were on ART and 85% were virologically suppressed [2].

Under the National AIDS Control Programme (NACP), CD4 count was estimated at periodic interval to monitor the progress of ART and HIV Viral load (VL) testing was done only among clinical failure and/or drug resistance cases. Based on World Health Organization (WHO) HIV VL testing guidelines, India has adopted VL testing for monitoring of PLHIV on ART. However, due to logistic constraints in terms of inadequate laboratory infrastructure and trained staff, there are challenges for implementing HIV VL testing in low and middle-income countries (LMIC) like India. Currently, VL testing samples from national programme are outsourced to private laboratories across the country. VL testing is not carried out on daily basis at ART centres (ARTC), making it difficult for the PLHIV to make up for the missed tests during their routine ARTC visits. This often ends up either in missing or postponement of VL testing among PLHIV and thus delays timely management of the treatment in case of virological failure. Hence there is a serious need for attempting other approaches for timely VL testing which can be implemented under the national programme. HIV VL reflects effectiveness of the antiretroviral therapy after treatment initiation and is helpful in diagnosing virological failures at the earliest. It also guides the clinician for adherence counselling and switching to next line ART drugs in case of virological failure [3]. Hence in the last few years, the HIV viral load testing was conducted through Public Private Partnership and during 2020–21, around 0.89 million VL tests were conducted through this venture [2].

Routine VL monitoring among PLHIV has favorable health outcomes than PLHIV monitored with CD4 testing alone. There are lower rates of loss to follow-up, death, and improved adherence is observed among PLHIV with regular VL monitoring. VL monitoring helps in identifying drug resistance and clinician to switch to second-line drugs if needed and thus improve the clinical outcomes [4]. Need for HIV viral load tests continues to increase up to 30 million by 2024 after adopting HIV viral load monitoring by point-of-care (PoC) technologies and dried blood spots (DBS) [5].

END AIDS 2030 target has now come up with a new set of targets achieving 95-95-95 percent each for HIV testing, treatment and virological suppression rates to be 95%—95%—95% by 2025. The last 95% of virological suppression is important in reducing HIV related morbidity and mortality, preventing development of drug resistance mutations, and significantly reducing HIV transmission [6]. Current VL assays require sophisticated laboratory facilities. As well as there are other challenges pertaining to insufficient laboratory access, shortcomings in the cold-chain management and sample transportation. In India, it has been estimated that only 7000 out of estimated 800000 tests are done annually [7], indicating need to explore other approaches for scaling up the HIV-1 viral load testing facilities.

With a view to address these inadequacies, the NACP has scaled up HIV-1 viral load testing across the country. The programme is now ready with 64 testing centers to which the peripheral ART centers are being linked up.

Several studies and an exercise commissioned by NACP to validate dried blood spot (DBS) as a sample type for VL testing has shown comparable viral loads using both sample types (Kulkarni et al [Unpublished]), suggesting that the existing VL infrastructure can be thoughtfully upgraded for considering DBS as an option for scaling up HIV-1 VL testing in hard to reach areas [8]. In comparison with plasma samples, DBS sample has added advantage pertaining to stability, no need of phlebotomy training and centrifuge facility. This added advantage will be helpful for ART centres especially Link ART centres (peripheral centres attached to ART centre) in resource limited settings like India. A systematic review and meta-analysis by Lara Vojnov et al assessed the technical performance of laboratory-based viral load technologies for accurate quantification of viral load using dried blood spot specimens compared to plasma specimens. While the performance varied between technologies, the most commonly used viral load technologies, Abbott RealTime HIV-1, bioMe´rieux NucliSENS EasyQ HIV-1, Roche COBAS TaqMan using the FVE protocol, and Siemens VERSANT HIV-1 RNA performed best and within acceptable limits when using a treatment failure threshold of 1,000 copies/ml. Study has recommended DBS specimen to improve access to viral load testing in resource- limited settings lacking the required infrastructure and cold chain storage for testing with plasma specimens [8]. To scale up HIV-1 VL testing centers in India, the independent evaluation of the Abbott HIV-1 VL kit using DBS as a sample type was carried out. It was found to be valid suggesting use of DBS based referred sample to improve HIV-1 VL testing accessibility in resource limited settings [9].

Additionally, to address these challenges especially in resource limited and remote communities, utilization of the PoC VL testing has been recommended [7, 10] suggesting that the GeneXpert platform PoC would be the best for ART management [11]. Many studies have reported that the plasma HIV-1 VL performed on platforms, the GeneXpert and HIV-1 Abbott m2000 are comparable and GeneXpert HIV-1 assay can be used as PoC for HIV-1 Viral load testing. Therefore, HIV viral load scale up using the existing GeneXpert PoC technology will also contribute in achieving the UNAIDS 95-95-95 target in our settings [12–14].

In India under National Tuberculosis Elimination Programme (NTEP), 1180 Cartridge Based Nucleic Acid Amplification Test (CBNAAT) facilities have been developed right from sub-district levels for diagnosing tuberculosis (TB) and detecting Rifampicin resistance. In 2017–18, about 2.4 million CBNAAT tests were performed and it was also utilized by nearly 0.6 million PLHIV for TB diagnosis [15, 16]. Despite of a huge network of CBNAAT testing, only 4% of TB/HIV co-infected individuals were tested using GeneXpert assay. HIV and Tuberculosis being a co-prevalent infection, there is need for integration of both tuberculosis and HIV services.

The GeneXpert HIV-1 VL assay has its own advantages in terms of simplicity of the assay, rapid turnaround time (90 minutes instead of 8 hours), compact equipment and cost effectiveness. Meta-analysis revealed that, GeneXpert HIV-1 VL performed well compared to current reference tests [17].

### Need of the current operations research

HIV-1 VL testing is a crucial part of HIV care and management and should be prioritized and scaled up at ARTC. In India, it is estimated that more than 800000 tests are needed annually, whereas only about 7000 are being done. This 90% testing gap can be addressed and minimized using suggested approaches. HIV-1 VL assay using GeneXpert platform requires around 90 minutes, which reduces the TAT as compared to the TAT for Abbott platform which is more than 14 days. Various studies have found concordance between GeneXpert and the VL testing by Abbott platform [High sensitivity (97%) / High specificity (97%)]. It is also established that a dynamic range of VL can be compared with GeneXpert (40−10^2^). The existing testing facility of CBNAAT can be leveraged for plasma HIV-1 VL testing which shall be essentially useful for both the program and people living with HIV. If found promising, these public health approaches can be helpful for further policy makers and program implementation.

In the remote areas where there is scarcity of both GeneXpert and Abbott platform, the DBS sample can be considered and transported by courier system for HIV-1 VL assay.

The key research questions are:

1. Can we integrate CBNAAT based facilities (GeneXpert assay) under NTEP to address the gaps in timely HIV-1 (VL) testing of PLHIV on antiretroviral treatment (ART) as per guidelines, in order to achieve the stated programmatic goals of VL testing?
2. Can we adopt dried-blood spots as a sample for HIV-1 VL testing to address gaps in VL testing of PLHIV on antiretroviral treatment (ART), in order to achieve the stated programmatic goals of VL testing?

The primary objective of the study is to assess the feasibility of integrating HIV-1 VL testing among PLHIV attending ART centres using the following public health approaches:

1. HIV-1 VL testing using GeneXpert based assay under NTEP (Model 1)
2. Use of DBS collected during routine visits at ARTC for VL testing at ICMR-NARI, Pune VL testing laboratory on Abbott platform (Model 2)

The secondary objectives of the study are:

1. To identify the gaps at the patient, service-provider, laboratory system levels during the implementation of two different public health approaches for HIV-1 VL testing as stated in objectives 1 and 2 as well as the existing system of using public/private laboratory providers.
2. To assess the acceptability of CBNAAT based HIV-1 VL testing among different Health care providers (HCP) after advocating the two public health approaches (Model 1 & Model 2)

## Methods

### Study design

This implementation research using mixed methods, will be conducted prospectively at ART centres in India.

### Study settings

India has implemented free ART services through a network of 620 ART centres to approximately 1.4 million PLHIV across the country. The current study will be done at selected ART centres across Indian states [3].

### Study intervention

The intervention models of VL testing will be based on the following strategies:

Model 1: CBNAAT platform based VL testing and validation on reference standard Abbott m2000 platform
Model 2: DBS based VL testing on Abbott m2000 platform

These two intervention models will be used as an approach for VL testing among PLHIV attending selected ART centres in the various states of India.

*1*. *Model 1 (CBNAAT platform based VL testing)*

**Fig 1:**
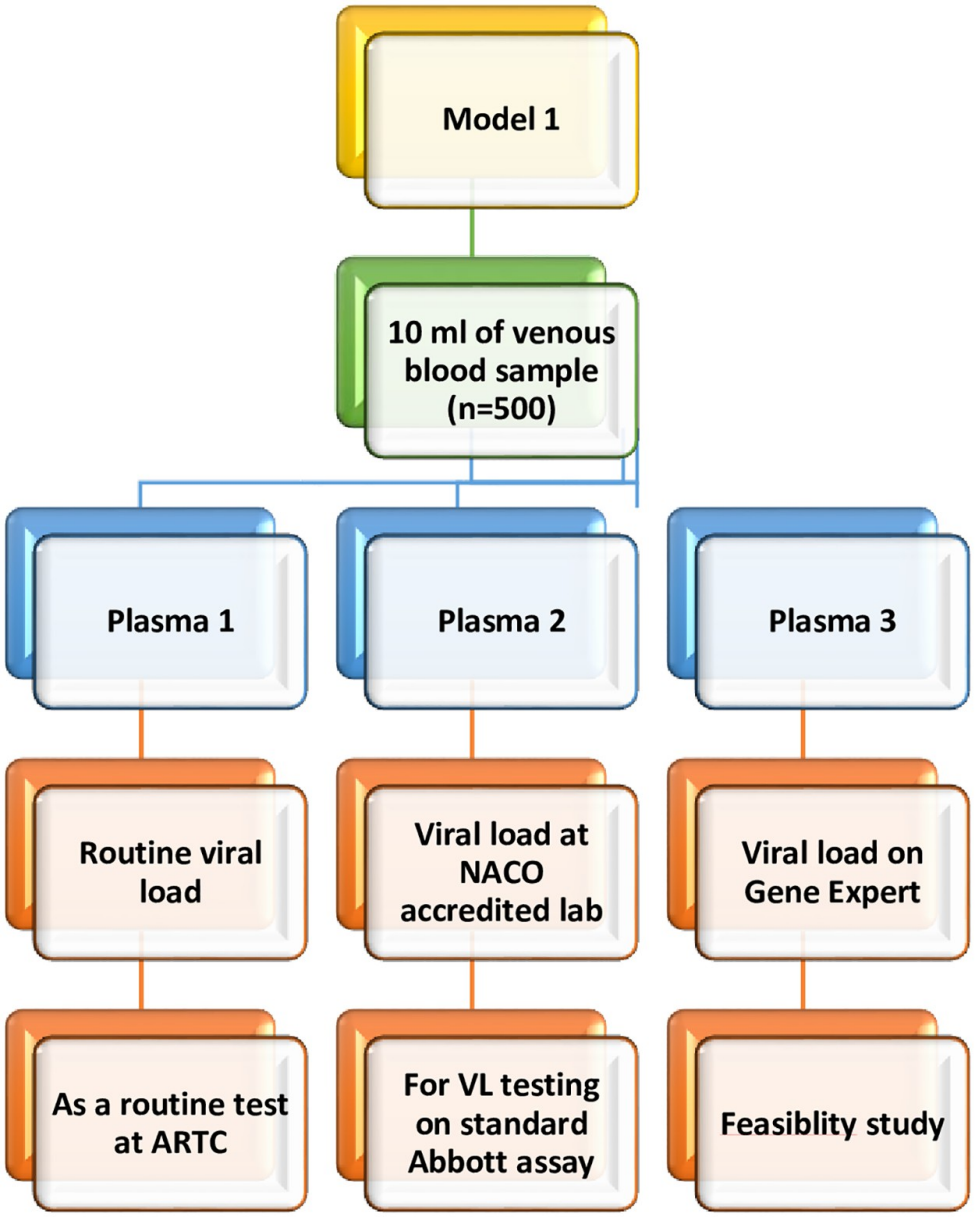
Flow diagram depicting intervention Model 1.

This model will leverage the existing CBNAAT centres for VL testing in the absence of Viral load testing centre (VLTC) in the same city or town where the ARTC is located (Fig 1).

We will test this strategy at one suitable ART centre from the selected state. In this model, plasma sample will be collected from the eligible consented PLHIV and transported to adjacent identified CBNAAT facility. The plasma sample will also be sent to an identified adjacent public/private laboratory for VL testing as a routine practice at ARTC under national programme. Additional plasma aliquots will be tested at NACO accredited reference laboratory for estimating VL on standard assay.

*2*. *Model 2 (DBS based VL testing)*

**Fig 2:**
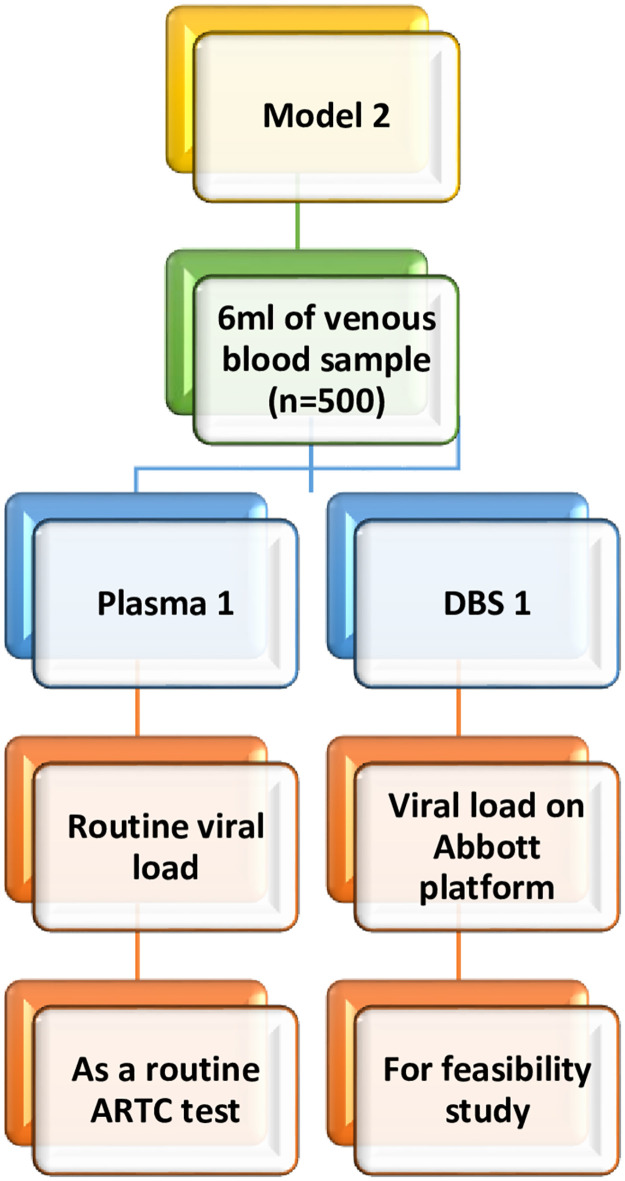
Flow diagram depicting intervention Model 2.

This model will adopt DBS sample for VL testing in absence of both CBNAAT and Viral load testing centre (VLTC) in the same city or town where the ARTC is located. Under this model, one DBS will be prepared from sample collected from eligible PLHIV and will be couriered to ICMR-NARI, Pune VL testing Apex laboratory (Fig 2).

The VL testing will be carried out using Abbott m2000 platform.

### Selection of study states

One or two states will be selected after reviewing the number of VL testing centres, PLHIV on ART and availability of CBNAAT facilities in the states. All the Indian states are categorized depending upon the number of VL testing centres available. Category 1 includes those states where there are more than four VL testing centres available, and Category 2 includes those states where 1–3 VL testing centers are available. Category 3 states are those where no HIV VL testing facilities are available. Categories 2 and 3 are considered for implementation of the study models. Of the category 2 (12 states), one ART centre will be selected for studying Model 1. In category 3 (12 states) with no VL testing centre (VLTC), one of the highest burden states will be selected for implementation of Model 2.

### Selection of ART centres

Model 1 will be implemented at one high burden ART center (more than 2000 registered PLHIV). Model 2 will also be implemented at one moderate to high burden ARTC as per discussion with NACO. As HIV-1 VL count done on CBNAAT platform needs to be compared with the standard laboratory assay, ARTC for Model 1 will be selected considering the feasibility of transporting plasma sample to nearby ICMR-NARI or NACO identified NABL accredited Public Sector Laboratory where Abbott platform for HIV-1 VL is available.

### Selection criteria for ART centre for implementation of Model 1

1. Non-availability of VLTC in same premises of the centre.
2. Availability of CBNAAT testing facility in the vicinity or in the same premises as ART centre.
3. Feasibility to transport plasma sample to NACO accredited VL testing laboratory (Abbott platform).

### Selection of CBNAAT facility for Model 1

1. Location of CBNAAT facility in the same vicinity or in the same town of selected ART centre.
2. Compatibility of CBNAAT facility for running HIV-1 VL test on plasma sample
3. Availability of CBNAAT platform for running additional HIV-1 VL test apart from routine TB testing.
4. CBNAAT facility will be finalized after discussion and approval from concerned state level TB stakeholders.

### Selection criteria for ART centre for implementation of Model 2

1. Non availability of VLTC in same city or town
2. Centre where the plasma sample is sent to NACO accredited lab for routine VL testing.
3. Non-availability of CBNAAT centre in same city or town *or*

Availability of CBNAAT facility with high testing burden (more than 3000 CBNAAT tests per year)

### Sample size

Being a feasibility study, sample size is not estimated for this study. However, it is anticipated that a total of 500 VL tests will be done at both the high burden ARTC and moderate burden ARTC during the 4 months enrollment period. Therefore, the estimated number of samples collected from each ARTC will be around 500 for each model, Model 1 and Model 2.

### Eligibility criteria

#### Participant inclusion criteria

Each individual must meet the following criteria to participate in the study:

1. PLHIV aged ≥18 years age.
2. Eligible for HIV-1 VL testing as per the guidelines.
3. Both follow up and newly registered PLHIV attending ARTCs during the enrollment period of the study.
4. Willing to give consent for the study.

#### Participant exclusion criteria

The following participants will be excluded from the study:

1. PLHIV aged < 18 years age.
2. PLHIV who are seriously ill, unwilling to provide consent for the study.

#### Participant recruitment and informed consent form for declaration from participant

The present study has obtained approval from the Institutional Ethics Committee (IEC) of the ICMR- National AIDS Research Institute, (Indian Council of Medical Research, Registration No: NARI-EC/2022-02) and informed consent will be obtained from each of the study participants before enrolling in the study. A participant information sheet (PIS) and Informed Consent Form (ICF) has been designed and approved by IEC. It consists of following statements:

- Statement that the study involves research and explanation of the purpose of the research
- Duration of the Study
- Description of the procedures to be followed, including all invasive procedures
- Type and quantity of biological sample collected
- Type of participants who will be involved in this study
- Statement on voluntary nature of participation in the study and right to refusal without any effect on receiving routine care and support at the ART centre
- Description of any benefits to the participant or others reasonably expected from research
- Description of any reasonably foreseeable risks or discomforts to the participant
- Statement describing the extent to which confidentially of records identifying the participant will be maintained and who will have access to participant’s medical records
- Reimbursement available to the participant to meet the expenditure for their visit
- Publishing study information for any scientific publication or presentation without revealing any personal identity
- Information about whom to contact for study related queries and rights of participant
- Informed consent form for declaration from participant.

### Data collection

A. A data abstraction sheet will be prepared to get the required information from each selected PLHIV from the selected ART centre. Nodal officer would be contacted and requested to provide the required information in the abstraction sheet provided. The information will include age, employment, duration since HIV infection and ART initiation, past clinical history, CD4 count, ART regimen, substitution and switches, drug adherence, history of tuberculosis (TB) and VL testing.

B. Sample collection and transportation:

#### Sample collection and transportation under Model 1 (n = 500)

Blood sample (10 ml) will be collected for VL estimation on GeneXpert CBNAAT platform (Model 1) from the PLHIV attending ART centre after seeking informed consent from the participant.

Plasma will be separated into three aliquots.

1. The first plasma aliquot will be sent to routine VL testing (public/private laboratories) as a part of ARTC routine activity.
2. Second plasma aliquot will be transported to the nearest CBNAAT testing centre for VL testing on the same day maintaining the cold chain. These samples will be transported along with the sputum or any other samples which are transported to the CBNAAT centres under NTEP.
3. Remaining plasma aliquot will be transferred to nearest NACO identified accredited reference laboratory for VL testing on the Abbott m2000 standard assay.

### Sample collection and rejection criteria for HIV-1 VL on Abbott platform

*Type of sample*:2*600 ul plasma specimens

*Type of container*: K2 EDTA as an anticoagulant

*Additive*: Blood will be collected in sterile vacutainers containing EDTA (violet top) or ACD (yellow top) as an anticoagulant.

*Sample rejection criteria*: Blood specimens with heparin as anticoagulant are unsuitable for this test. Plasma specimens collected from hemolyzed, contaminated, very lipemic blood are unsuitable for this test.

### Sample transportation

The biological samples will be transported as per the current regulations. Whole blood is processed for plasma separation within 6 hours of sample collection. Separated Plasma specimens of various peripheral clinics will be transported at 2–8°C or frozen at -70°C.

### Sample specimen processing and storage

Plasma will be separated from whole blood. Plasma specimen will be stored at 15–30°C for up to 24 hours or at 2–8°C for up to 5 days. If required to be stored for longer duration, it will be kept at -70°C. Multiple freeze thaw cycles will be avoided. The frozen specimens will be thawed at 2–8°C. Once thawed, plasma sample may be stored at 2–8°C for up to 6 hours. As it is not possible to retain the extracted RNA from automatic sample processing m2000sp Abbott machine, plasma samples for HIV-1 VL have to be retained up to four months after the date of report dispatch. The plasma sample aliquot will be stored at -70°C or below till the time of discarding.

### GeneXpert HIV-1 viral load assay

GeneXpert Instrument System has the automated and integrated mechanism for sample preparation, nucleic acid extraction, amplification, and detection of the target sequence in plasma samples using real-time PCR. The integrated system consists of an instrument component and a dedicated computer with preloaded software for running the test samples and verifying the final results. The systems require the single-use disposable GeneXpert cartridges that hold the assay reagents to carry out the processing of RNA extraction followed by the qRT-PCR.

The HIV-1 GeneXpert Viral Load tests will be performed on the available GeneXpert platform along with TB testing simultaneously, while prioritizing the test for TB. The whole blood samples will be collected, and the plasma will be separated for HIV-1 Viral Load testing. The isolated plasma samples will be loaded onto the GeneXpert cartridges and tested for HIV-1 viral load. The results will be recorded after the overall run time of 90 minutes for the instrument. To assess the feasibility of GeneXpert platform, the data will be collected on pretested questionnaire pertaining to the number of samples tested for HIV-1 Viral Load, and the number of samples tested for TB.

### The Abbott real time HIV-1 assay

It is an in-vitro nucleic acid amplification test that uses exponential amplification and real time detection of HIV-1 RNA in human plasma. Abbott m2000SP and m2000RT platforms are automated platforms which need the kits specified by the manufacturer for RNA extraction, cDNA synthesis and amplification The Abbott m2000rt machine automatically gives the results on Abbott m2000rt workstation after completion of real time PCR assay.

### Sample collection and transportation under Model 2

Blood samples will be collected after assessing eligibility and administration of informed consent form. From the collected blood sample, one DBS card will be prepared, and plasma aliquot will be sent to routine VL testing (public/private laboratories) as a part of ARTC routine activity. The DBS sample will be transported to ICMR-NARI, Pune on a weekly basis and will be processed for VL testing on the Abbott m2000 platform at ICMR-NARI, Pune.

### Protocol for HIV-1 DBS VL on Abbott m2000 platform

#### Sample preparation or preparation of DBS

The DBS cards will be prepared by spotting 70ul whole blood onto a Whatman 903 filter paper.

#### Sample transport

Filter papers will be air dried overnight at room temperature. The DBS cards will be transported at regular intervals in plastic sealed bags along with a desiccant and a humidity card at room temperature to ICMR-NARI.

#### Sample processing

The HIV-1 viral load will be estimated at ICMR-NARI as per Abbott manufacturer’s instructions.

Sample transportation log will be maintained for each model to estimate Turnaround time (TAT). TAT for sample transportation, sample processing and dispatch of results will be estimated. Viral load results obtained on different assays will be captured in a predesigned study tool.

In a summary, data collection will collect following information:

### Data collection forms at ART centre level

- Participants’ socioeconomic information
- Personal history, family history, medical history
- Information pertaining to HIV diagnosis and ART initiation
- Clinical assessment from the records
- Laboratory assessment from the records

### Details of sample collection under study

#### Data collection forms at Laboratory level during different assays

C. In order to assess the feasibility of implementation of different models, in-depth interviews (IDI) will be conducted among the ARTC lab technicians, CBNAAT lab technicians, laboratory technicians in VLTC and Medical officers of ART centres after obtaining informed consent. Program managers and officials will also be interviewed for addressing any issues regarding the model implementation. IDIs will also address the approaches currently used for communication of HIV VL test results and need of any other approaches in order to reduce TAT. All qualitative IDIs would be conducted in local language by the local study team in person or through online mode and audio recorded. IDIs will follow a semi-structured format, covering a set of topics listed in a pre-established topic guide, with the flexibility of encouraging participants to contribute additional insights. Each IDI is expected to last for a maximum of 60 minutes. Subsequently these would be transcribed as exact verbatim and then translated in English. All interviews will be conducted in the clinic or at a place convenient to the participants where confidentiality will be taken care of. The interviews will be digitally recorded. The IDI guide will be prepared, and themes related to the questions related to experiences, challenges and facilitators of care among PLHIV will be identified.

### Data analysis

Data from different centres would be entered in Microsoft excel 2013. The clinical and social characteristics of study participants will be analyzed. Association between variables will be determined using odds ratio and 95% CI. Software package SPSS 20.0 will be used for data analysis. Comparative analysis of VL detected on different assays will be done. The VL values (copies/mL) will be transformed to log10 copies/mL. The concordance proportion will be derived for VL testing based on standard assay against CBNAAT assay. Concordance will also be calculated for VL testing on DBS sample on standard platform and plasma samples on CBNAAT platform. Limits of agreement and mean bias (95% CI) will be studied using the Bland-Altman plot. The misclassifications rates for different VL cut-offs will also be statistically tested.

The proportion of PLHIV tested under each study model will be compared with proportion of PLHIV tested under current ART centre practices. Similarly, TAT pertaining to testing, transportation and dispatch time under each study models will be compared with standard ART VL testing practices.

As per NACP-V guidelines (2021) published by NACO [3], the virological suppression in context of the national programme means a plasma viral load of less than 1000 copies/ml after at least 6 months on ART. Hence, the <1000 copies/ml threshold will be used for considering virological suppression.

The analysis will be done to derive the following study indicators as shown in Table 1.

**Table 1:** Outcome indicators of the study.

Study Indicators for Model 1	Study Indicators for Model 2
1. Proportion of PLHIV tested for VL at ARTC under model 1: *The proportion of PLHIV tested for VL at ARTC under model 1 denotes the percentage of PLHIV whose HIV- VL test was due as per the current national programme guidelines and were tested for VL testing under model 1.	1. Proportion of PLHIV tested for VL at ARTC under model 2: *The proportion of PLHIV tested for VL at ARTC under model 2 denotes the percentage of PLHIV whose HIV- VL test was due as per the current national programme guidelines and were tested for VL testing under model 2.
2. Proportion of PLHIV tested for VL at ARTC as per standard ART centre practices: *The proportion of PLHIV tested for VL at ARTC as per standard ART centre practices denotes the percentage of PLHIV whose HIV- VL test was due as per the current national programme guidelines and were tested for VL testing as per current ART centre standard practices.	2. Proportion of PLHIV tested for VL at ARTC as per standard ART centre practices: *The proportion of PLHIV tested for VL at ARTC as per standard ART centre practices denotes the percentage of PLHIV whose HIV- VL test was due as per the current national programme guidelines and were tested for VL testing as per current ART centre standard practices.
3. Total turnaround time (TAT) for VL testing Model 1 approach including TAT for sample transportation, sample testing and receipt of results	3. Total turnaround time (TAT) for VL testing Model 2 approach including TAT for sample transportation, sample testing and receipt of results
4. Total turnaround time (TAT) for each VL testing including TAT for sample transportation, sample testing and receipt of results as per the current standard practices at ART centre.	4. Total turnaround time (TAT) for each VL testing including TAT for sample transportation, sample testing and receipt of results as per the current standard practices at ART centre.
5. Proportion of CBNAAT TB tests and HIV-1 VL tests	5. Proportion of PLHIV with virological suppression
6. Ratio of HIV-1 VL and CBNAAT TB test per assay in the CBNAAT centre over time	6. Proportion of sample rejections and reasons for the same
7. CBNAAT samples tested under NTEP before and after implementation of Model 1.	
8. Proportion of PLHIV with virological suppression	
9. Proportion of sample rejections and reasons for the same	
10. TAT for the sputum samples tested for CBNAAT under NTEP	
Correlation coefficient between DBS based and plasma based VL testing	

### Data safety and confidentiality

The study participants will be assigned a unique identification number (ID) to be used in the study database. The document linking the IDs to the names of study participants will be kept in a locked office, will not be accessible to personnel not associated with the study. The Data Entry Operator and the Study Statistician will check for data completeness and accuracy.

### Dissemination of results

The results of the research protocol will be communicated with the stakeholders of NACP and NTEP through personal meetings. A technical brief will also be prepared to inform policy makers to improve accessibility of HIV VL testing based on study findings. Study results will also be published in different scientific literature and presented in various national and international conferences.

## Discussion

This project will provide knowledge regarding the feasibility of PoC GeneXpert platform and dried blood spots for HIV-1 VL testing among people living with HIV attending ART centres in India. The public health approaches will help in adapting new contexts and implementation in the national HIV programme settings. There is a need to adapt, integrate, and optimize these approaches for VL testing as India marches towards achieving Fast track targets of 90-90-90 which has now targeted as 95-95-95 and assists in ending HIV as public health threat by 2030.

### Expected outcome

The current implementation research will focus on assessing the feasibility of integrating HIV VL testing among PLHIV attending ART centres using various public health approaches. The proposed outcome indicators are summarized in Table 1.
